# Comparative Psychology: A Perspective Rather than a Discipline. Commentary: A Crisis in Comparative Psychology: Where Have All the Undergraduates Gone?

**DOI:** 10.3389/fpsyg.2015.01828

**Published:** 2015-11-25

**Authors:** Cinzia Chiandetti, Walter Gerbino

**Affiliations:** Psychology Unit, Department of Life Sciences, University of TriesteTrieste, Italy

**Keywords:** comparative psychology, mind-brain, discipline, perspective, undergraduates, recruitment, cognition-emotion, comparative cognition

In the Twentieth Century animal behavior was studied by European ethologists and American behaviorists. By fusing these approaches, comparative psychology became the framework for addressing animal behavior issues and understanding psychological functions shared by a variety of species. Relying on interspecies comparisons, comparative psychology complements developmental psychology and differential psychology, which are focused on intraspecies comparisons. All are better conceived as integrable perspectives, we think, rather than disciplines.

Abramson (2015) describes an academic crisis that is emphasized (if not induced) by his personal way of contrasting behavior vs. cognition, grounded on the identification of psychology with the study of behavior and the assumption that the study of cognition is based on “suppositions” and “beliefs” (in Abramson's words). We disagree with both grounding propositions. Behavior of organisms is studied by several disciplines. What makes psychology special is the application of the scientific method to the study of mental phenomena (as they appear to individuals), as well as mental constructs (as elements of a theory). Cognitivism produced *models* and *evidence* to explain cardinal phenomena missed by radical behaviorism. For instance, in classical conditioning the conditioned stimuli must be predictive of the unconditioned stimuli for conditioning to occur (Rescorla, 1988); namely, the mere contiguity between stimuli is not sufficient. Furthermore, the study of behavior in controlled settings cannot be the defining feature of comparative psychology only, given that it is shared with other disciplines (behavioral economics, microsociology, behavioral neuroscience).

Abramson seems to underestimate the need for psychology—to hold the promise embodied in its name—to take behaviors (of human and nonhuman animals in their natural environments or in constrained settings, of brain areas, of single neurons) as observables that carry information about its *explananda*, which in the words of James (1890, *Preface*) are “thoughts and feelings.” Cognition and emotion are the indispensable objects of psychological research, to be studied through the *analysis* and *comparison* of behaviors and explained, as regards proximate causes, by relevant neural mechanisms. Reference to functions provides animal behaviors and brain activations with meaning, as expected by most people including undergraduates.

However, Abramson's dissatisfaction does not match the point of view reflected in a recent report of the Comparative Cognition Society (Weisman et al., 2015), the articulation of APA, and authoritative evaluations of current comparative biology (Bateson, 2012). This point of view sees the future of behavioral sciences in the integration with other disciplines.

Abramson's pessimism might be the consequence of the dominance of neural explanations (referring to proximate causes) over biological explanations (referring to the phylogenesis and ontogenesis of cognition and emotion). We are convinced that a comparative perspective on functions and mechanisms (not behaviors) can effectively balance neuroscience, like in the following conclusion on visual cognition: “whether an animal is using a collothalamic- (birds) or lemnothalamic-dominant (mammals) visual system, they may operate using similar computational and processing principles because of the structure of the visual world” (Qadri and Cook, 2015). Significantly, this conclusion comes from a laboratory that approaches questions from the comparative cognition perspective and looks beyond neural differences to identify the role of environmental constraints in shaping a function. Even when only differences and no similarities are available (a limiting case in the comparative arena) still a *Gedankenexperiment* on how the same function might appear in another animal, in a different way, can provide an insight about its nature.

The work by O'Keefe and M.-B. and E. Moser acknowledged with the Nobel Prize in 2014 helped in deepening the understanding of the mechanism for spatial navigation (O'Keefe, 1971; Hafting et al., 2005). However, such physiological studies resolve both scientific and philosophical questions only when integrated with investigations on the utility and the development of the mechanism (Wills et al., 2010; Bjerknes et al., 2014), its evolution (Bingman and Sharp, 2006; Yartsev et al., 2011) and the nature/nurture related aspects (Dehaene et al., 2006; Chiandetti et al., 2015). Furthermore, when supported by computational models, the research can profit of simulations that sometimes even anticipate evidence coming from real organisms (Burgess et al., 2000; Urdapilleta et al., 2015).

Integration of research efforts converging on spatial navigation represents a successful example of the application of Tinbergen's fruitful warning. In his seminal work (1963) Tinbergen suggested that true understanding of complex functions requires an integrated approach within which—we believe—the comparative psychology perspective can link apparently distant fields (Tinbergen, 1963).

Within most academic institutions comparative psychology will better act as one of the fundamental perspectives than presenting itself as a leading discipline in a dedicated undergraduate program. Undergraduates enrolled in several programs—including psychology and biology, of course—should be exposed to the comparative perspective to develop an integrated understanding of mind-brain systems.

Tinbergen's warning should be taken more broadly into account (Bateson and Laland, 2013) and should motivate the convergence of different areas of expertise. Heterogeneous teams composed of people with different backgrounds are probably the most fecund, especially with the rate of change in technological innovations that need to be creatively adapted to the study of minds/brains of disparate species. Diversity fosters knowledge.

As ethology and behaviorism molded together to support a more exhaustive and controlled study of animal behavior, comparative psychology should complement other perspectives and intersect various levels of analysis (neural, genetic, ecological, evolutionary) to answer all Tinbergen's questions. The full understanding of cognition and emotion will take place in a nameless neutral field, fed by specialized disciplines but a step away from them.

## Author contributions

CC and WG discussed the topic, contributed to the writing of the manuscript and approved its final version for submission.

### Conflict of interest statement

The authors declare that the research was conducted in the absence of any commercial or financial relationships that could be construed as a potential conflict of interest.
